# Mechanism and inhibitory effects of cactus (*Opuntia dillenii*) extract on melanocytes and its potential application for whitening cosmetics

**DOI:** 10.1038/s41598-022-26125-x

**Published:** 2023-01-10

**Authors:** Chien-Shan Chiu, Yu-Tsung Cheng, Yung-Jia Chan, Wen-Chien Lu, Kai-min Yang, Po- Hsien Li

**Affiliations:** 1 Department of Dermatology, Taichung Veterans General Hospital, 1650 Sec. 4 Taiwan Boulevard, Xitun Dist., Taizhung, 40705 Taiwan; 2 Institute of Biomedical Sciences, National Chung Hsing University, 145 Xingda Rd., South Dist., Taizhung, 40227 Taiwan; 3 Department of Medicinal Botanical and Foods on Health Applications, Da-Yeh University, No.168, University Rd., Dacun, Changhua, 51591 Taiwan, ROC; 4 Cardiovascular Center, Taichung Veterans General Hospital, 1650 Sec. 4 Taiwan Boulevard, Xitun Dist., Taizhung, 40705 Taiwan; 5 College of Biotechnology and Bioresources, Da-Yeh University, 168, University Rd, Dacun, Changhua, 51591 Taiwan; 6 Department of Food and Beverage Management, Chung-Jen Junior College of Nursing, Health Sciences and Management, 217, Hung-Mao-Pi, Chia-Yi City, 60077 Taiwan; 7 Department of Food Science, National Quemoy University, 1, University Rd., Jinning Township, Kinmen County, 892 Taiwan; 8 Department of Food and Nutrition, Providence University, 200, Sec. 7, Taiwan Boulevard, Shalu Dist., Taizhung City, 43301 Taiwan

**Keywords:** Plant sciences, Health care

## Abstract

Penghu cactus (*Opuntia dillenii* [Ker.] Haw) is a cactus plant that commonly grows in Penghu Island, Taiwan, Republic of China (ROC). However, still lack of scientific study on the *Opuntia dillenii* [Ker.] Haw extract on skin-whitening-associated tyrosinase activity and melanin production. The activities of its extract in melanogenesis were investigated in this article. In this experiment, we used an extract from the Penghu cactus (*Opuntia dillenii* [Ker.] Haw) to study its tyrosinase inhibition, anti-melanin generation, UV-protection effects and wound healing capacity in B16-F10 melanocytes. Without reducing cell growth greatly or causing cell death, 20 g/L cactus extract effectively inhibited the melanin production of B16-F10 cells, and melanogenesis was induced by 3-isobutyl-1-methylxanthine. The cactus extract could also promote cell proliferation. Cactus extract treatment decreased the mRNA expression of insulin-like growth factor 1 (IGF-1) and vascular endothelial growth factor (VEGF) and increased that of transforming growth factor β (TGF-β). Thus, it could reduce cell melanin production and promote cell growth but by also reducing IGF-1 and VEGF mRNA expression, may reduce wound scarring and prevent tumor proliferation and swelling. Increasing TGF-β mRNA expression can help increase collagen to remove wrinkles and help in wound healing. Skin patch test results agreed with in vitro results with B16-F10 melanoma cells. The cactus extract significantly inhibited tyrosinase activity and reduced melanin production, showing a whitening effect on skin tests. Cactus may be a good natural candidate for inhibiting melanin production and promoting cell proliferation.

## Introduction

Melanin pigments are synthesized from a protective mechanism, called melanogenesis, to protect the skin from ultraviolet radiation^1^. However, abnormal production or accumulation of melanin will be causing pigment disorders, for example, melasma (chloasma), hyperpigmentation, lentigines, freckles, and ephelides^2^. Even though skin color is affected by congenital genes, nevertheless, sun exposure, hormone changes, and food intake greatly influenced the melanogenesis of melanocytes^3^. Tyrosinase is a key enzyme that plays a vital role as a rate-determining step enzyme which involved in melanogenesis to generate melanin. Moreover, skin diseases are also closely related to tyrosinase activity and melanin production. This enzyme is mostly found in living organisms, for instance in plants, animals, bacteria, and fungi, which also play a vital role in the browning process^4^.

Most drugs available in the market are chemosynthetic drugs and come across with side effects on the patient during and after the treatment, especially the drugs used to treat cancer^5^. To overcome the side effects, naturally obtained drugs from medicinal plants are preferred. Traditional medicine has a long history that begins with the look for botanicals to heal various diseases^6^. The *Opuntia* (*Opuntia spp.*) is a cactus plant that commonly grows in almost all climates, such as wild, temperate, and tropical climates^7^. The bioactive compounds and biological activities of *Opuntia spp.* usually altered by the environment and growing conditions^8^. Therefore, there is an opportunity for the study of different functioning properties from different *Opuntia spp.* cultivars. *Opuntia spp.* not only contained sugar, fatty acid, tocopherols, and organic acid, but also flavonoid glucosides, kaempferol, lutein, and β-carotene^9^. In addition, with the presence of phenolic and flavonoids, previous studies reported the high antioxidant capacity, lower cholesterol level, anti-inflammatory activities, and enhanced wound healing capabilities of *Opuntia spp.*^10,11^*.* Moreover, cactus peel (*Opuntia* peel) which makes up nearly 60% of the whole cactus fruit is regarded as a useful by-product^12^. The *Opuntia* peels are rich in dietary fiber and contained polysaccharides, vitamins, and flavonoid glycosides as the major flavonoids profile^13^. The extract of *Opuntia* demonstrated a significant antioxidant activity, upsurge the excretion of cholesterol and lower the liver cholesterol level^14^. Meanwhile, the reports of anticarcinogenic, anticancer, and antihypertension effects also highlight the *Opuntia* peel as a high-value food ingredient^15^. Previous studies also demonstrated that ethanol extracts of the *Opuntia dillenii* [Ker.] Haw were found to exhibit efficient antilarval efficacy against the larvae of *Aedes aegypti* (with LC_50_ value of 246.1 µg/mL), and anticancer efficacy against HeLa cancer cell lines (inhibition percentage with the IC_50_ value of 73.48 µg/mL)^16^. Furthermore, *Opuntia dillenii* [Ker.] Haw which has higher phenolic content and antioxidant power indicated a significant increase in motility in human sperm quality after a freeze–thaw cycle^17^.

Despite its widespread use, to the best of our knowledge, to date, still a lack of scientific study on the *Opuntia dillenii* extract on skin-whitening-associated tyrosinase activity and melanin production. Nevertheless, hypotensive^18^, analgesic^19^, potential antibacterial and antifungal^20^, radical scavenging^21^, and anti-spermatogenic effect^22^ of *O. puntia* have been reported. Hence, in this study, the tyrosinase inhibitory activity and melanin production of the *O. dillenni* was examined. To address the inhibitory activity, the effects of *O. dillenni* on 3-isobutyl-1-methylxanthine (IBMX) induced melanogenesis and tyrosinase inhibition in B16-F10 murine melanoma cells were assessed. In the meantime, the mRNA expression of insulin-like growth factor (IGF-1), transforming growth factor (TGF-β), and vascular endothelial growth factor (VEGF) was also determined to evaluate the wound healing capacity of *O. dillenni* extract.

## Material and methods

### Materials

Dulbecco’S Modified Eagle’s Medium (DMEM); Fetal Bovine Serum (FBS); Penicillin–Streptomycin (PS); 3-Isobutyl-1-methylxanthine (IBMX); Methyl 3,4,5-trihydroxybenzoate (Methyl gallate, MG); Trypan blue; Tetrazolium violet; All chemicals used in this study were American Chemical Society (ACS) certified of analytical grade.

### Cell strains

B16-F10 cell line: *Mus Musculus* skin melanoma is an adherent cell that can produce melanin; purchased from Bioresources Collection and Research Center (BCRC 60031). B16-F10 is a mixture of spindle-shaped and epithelial-like cells, which are incubated at a temperature of 37 °C with a 5% of carbon dioxide concentration. While the culture medium was prepared by 90% Dulbecco's modified Eagle's medium (DMEM) with 4 mM L-glutamine adjusted to contain 1.5 g/L sodium bicarbonate and 4.5 g/L glucose + 10% fetal bovine serum (10,437–028, GIBCO, Carlasbad, USA).

### Sample preparations

**Figure 1:**
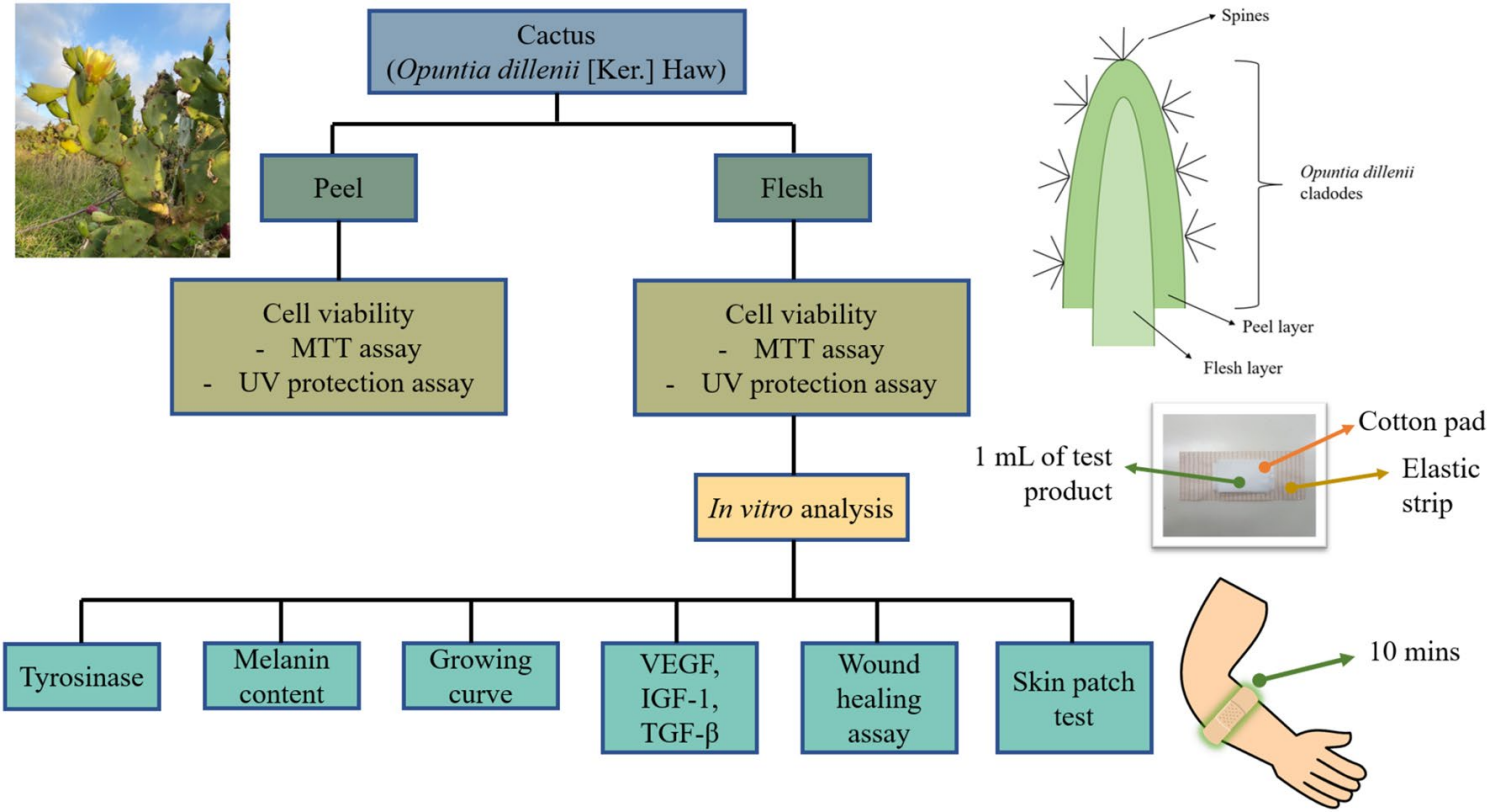
The experimental design of cactus extract (peel and flesh) on B16-F10 cell proliferation and tyrosinases inhibition effect.

The cactus (*O. dillenii* [Ker.] Haw) used in this experiment was provided by Sea Mild Biotech. (Taoyuan City, Taiwan). We selected fresh mature *Opuntia* cladodes with an average size of 23 cm. After washing and cleaning, the spine of the cactus was removed, and the peel and flesh layer were separated. Then 100 g cactus peel together with 1 L pure water was homogenized by using a homogenizer (N1611, IUL Masticator Basic (400 ml) Analog Lab Blender, Spain). The mixture was sterilized (TM-321, Tomin Autoclave, Taiwan), and we finally obtained 1 L of cactus peel extract. The extractant was filtered and the extract was then dried by a vacuum-freeze drier (FDM-5, UNISS, Taiwan) for 48 h. For the following sample, the spine and outer peel of the cactus were removed, next, 100 g of cactus flesh together with 1 L of pure water was homogenized by using a homogenizer (N1611, IUL Masticator Basic (400 ml) Analog Lab Blender, Spain). Then the mixture was sterilized by sterilizer (TM-321, Tomin Autoclave, Taiwan), and finally obtained 1 L cactus extract of flesh extract. The extractant was filtered and the extract was then dried with a vacuum-freeze drier (FDM-5, UNISS, Taiwan) for 48 h. The dried extract was collected and stored in a freezer with a double-bagged polyethylene. Figure 1 demonstrated the experimental design of cactus extract on B16-F10 cell proliferation and tyrosinases inhibition effect.

### Determination of cell viability by MTT assay

Cell viability was assessed by MTT method of Chan et al. (2011) with modifications^23^. B16F10 cells were incubated in a 96-well plate at a density of 5 × 10^3^ cells/well for 24 h. Next, the cactus sample with different concentrations (0, 0.625, 1.25, 2.5, 5, 10, and 20 g/L) was added, and the cells were incubated for 48 h at 37 °C under 5% of CO_2_. After centrifugation, the supernatant was discarded, 20 μL of 5 mg/mL MTT was added, then continued incubation under the same condition for another 3.5 h. The MTT was discarded and followed by adding 150 µL of MTT solvent. The absorbance was determined at a wavelength of 590 nm by using the microplate reader (Thermo Scientific, America).

### Ultraviolet protection assay

One hundred μL of B16-F10 cell line with a concentration of 5 × 10^3^ cells/mL were cultured into 96 wells plate and incubated at 37 °C for 24 h. Next, the control sample (cells without extract), 20 g/L cactus peel extract, and 10 g/L cactus flesh extract, together with vitamins C and E, respectively, were irradiated under ultraviolet light for one hour. After culturing for 24 h, the supernatant was removed, 20 μL of 5 mg/mL MTT was added directly and placed in a 37 °C incubator for 3.5 h. After that, MTT was removed, 150 μL of MTT solvent was added, and a 96-well plate was wrapped with aluminum foil and placed on a rotary shaker for 15 min. Finally, the absorbance was measured at a wavelength of 590 nm.

### RNA extraction

The cells were placed in a 1.5 mL microcentrifuge tube and centrifuged at 2000 rpm for 5 min. Afterward, the medium was removed, and 1 mL of RNA extract and 200 μL of Chloroform were added in and continued to mix vigorously. Next, the mixture was centrifuged at 12,000 rpm for 15 min to make it separated into three different layers, which are the water layer containing RNA at the top, the white middle layer containing DNA and protein, and the lower organic solution layer. The water layer was transferred to a 1.5 mL microcentrifuge tube, and an equal volume of isopropanol was added in, then continued to mix it well. The tube was placed in a − 80 °C refrigerator for 30 min to facilitate the precipitation of RNA. The RNA at the bottom of the centrifuge tube was concentrated by centrifugation (12,000 rpm, 10 min, 4 °C), then added in 1 mL of 75% DEPC-Ethanol after removing the supernatant, and continued with centrifugation (12,000 rpm, 10 min, 4 °C). The supernatant is removed again, and the alcohol is evaporated and dried. Finally, 20 μL of DEPC-H_2_O is added to dissolve the RNA, and the integrity of the RNA is confirmed by electrophoresis with 1% Agarose Gel. To determine its concentration, 1 μL of RNA was added to 999 μL of pure water (diluted 1,000 times). O.D. 260 nm was used to measure RNA content, while protein content was measured at O.D. 280 nm. The O.D. for RNA determination is 260 nm/O.D. 280 nm. The ratio must be between 1.6 and 1.8, and the RNA concentration (μg/mL) calculation formula is O.D. 260 nm × 40 × dilution factor.

### RNA electrophoresis

0.2 g of agarose powder and 42 mL 0.5 × TBE buffer were added into an Erlenmeyer flask and heated up until the powder was dissolved completely. The dissolved agarose was poured into the gel tray where the gel comb has been inserted and waiting for the agarose to solidify before pulling out the gel comb. The solidified agarose gel was put in the electrophoresis tank, and 0.5 × TBE buffer was poured to cover the gel. After mixing the loading dye and RNA sample, injected into the comb slot, and connected the negative electrode to the end of the colloid hole and the positive electrode to the other end, while the electrophoresis was performed at 100 V.

### Reverse transcription polymerase chain reaction, RT-PCR

Five μL RNA and 1 μL 10 μM Random Hexamer Primer was added into the nuclease-free PCR reaction tube and replenished the DEPC-H_2_O to 14 μL. Next, the reaction tube was put in a PCR reactor, and the reaction was at 65 °C for 15 min to denature the RNA secondary structure. After 15 min, the reaction tube was taken out and placed directly on ice for 5 min to prevent the recovery of RNA secondary structure. The reaction continued by adding 5 μL 5 × FS Buffer, 2.5 μL DTT (0.1 M; Dithiothreitol-01), 2 μL dNTP (2.5 mM), 0.5 μL RNaseOUT (40 U/μL) into the reaction tube and put it back in the PCR machine. After reaction at 37 °C for 5 min, 1 μL Moloney Murine Leukemia Virus (M-MLV) was added in, mixed up thoroughly, and put back into the PCR machine with the setting conditions at 25 °C (10 min), 37 °C (90 min), 70 °C (15 min), finally cooled to 4 °C and stored at − 20 °C.

### Targeted mRNA expression

#### Control sample

The cDNA used glyceraldehyde-3-phosphate dehydrogenase (GAPDH) as an internal control group. Six μL pure water, 10 μL 2 × Taq DNA Polymerase Master Mix Red (Ampliqon, 180301, skovlunde, Denmark), 2 μL cDNA, 2 μL GAPDH primer mix (10 μM forward, 10 μM reverse) in a PCR reaction tube, the conditions were set 5 min at 94 °C, followed by 25 cycles of 94 °C for 30 s, 55 °C for 30 s, 72 °C for 30 s, and 72 °C for 10 min. Finally cool down to 4 °C. The products after the reaction were separated by 1% agar gel electrophoresis, and the expected amplified fragment of GAPDH was 266 base pairs (bp).

#### Tyrosinase

The sample for Tyrosinase fragment amplification is the same as that in Sect.  “Control sample”. The setting conditions are 94 °C for 5 min, followed by 30 cycles of 94 °C for 30 s, 55 °C for 30 s, 72 °C for 30 s, and 72 °C for 10 min. Finally cool to 4 °C. The products after the reaction were separated by 1% agar gel electrophoresis, and the expected amplified fragment of Tyrosinase was 220 bp.

#### Vascular endothelial growth factor (VEGF)

The sample of VEGF fragment amplification is the same as mentioned in Sect.  “Control sample”. The setting conditions are 94 °C for 5 min, followed by 94 °C for 30 s, 58 °C for 30 s, 72 °C for 30 s, and the reaction is 30 cycles, 72 °C for 10 min. Finally cool to 4 °C. After the reaction, the products were separated by 1% agar gel electrophoresis, and the expected amplified fragment of VEGF was 127 bp.

#### Insulin-like growth factor (IGF-1)

The sample of IGF-1 fragment amplification is the same as that in Sect.  “Control sample”. The setting conditions are 94 °C for 5 min, followed by 94 °C for 30 s, 60 °C for 30 s, 72 °C for 30 s, and the reaction is repeated for 30 cycles, and 72 °C for 10 s. Finally cooled to 4 °C. The products after the reaction were separated by 1% agar gel electrophoresis, and the expected amplified fragment of IGF-1 was 401 bp.

#### Transforming growth factor beta (TGF-β)

The sample of TGF-β fragment amplification is the same as that in Sect.  “Control sample”. The setting conditions are 94 °C for 5 min, followed by 94 °C for 30 s, 60 °C for 30 s, 72 °C for 30 s, 28 cycles of reaction, 72 °C for 10 s, and finally cooled to 4 °C. The products after the reaction were separated by 1% agar gel electrophoresis, and the expected amplified fragment of TGF-β was 300 bp.

### Growing curve

The cells were counted as 2 × 10^4^ cells/well in 12 wells. After culturing in a 37 °C incubator for 24 h, 20 g/L of the cactus extract was added, and the number of cells was counted in one grid every day, and the number of cell growth was recorded. First, added in 500 μL of PBS to wash the well. Next, removed the PBS, and added 250 μL of Trypsin to detach the cells. Then continued by adding 250 μL of Medium. 10 μL of the mixture was added to 10 μL of 0.4% Trypan blue and mix well. Finally, take 10 μL of the mixture into the hemocytometer, and counted using an upright microscope.

### Melanin content determination

Cells with a concentration of 1 × 10^5^ cells/well were pre-cultured in 6 wells of a 96-wells plate, and cultured in a 37 °C incubator for 24 h. Next, 7 μL/mL IBMX (0.1 mM) and 20 g/L cactus extract were added in, respectively. After induction for 2 days, the cells were detached with Trypsin, and collected, followed by centrifugation (6000 rpm, 2 min). The supernatant was removed, 250 μL of 1 N NaOH was added, and the cells were heated to 100 ˚C in boiling water for 1 h. After 1 h of heating, Vortex mixing for 15 secs, and absorbance was measured at OD450 nm^23^.

### Wound healing assay

Pre-incubated 1 × 10^5^ cells/well in 6 wells and cultured in a 37 °C incubator for 24 h. A gap was drawn in each well by using a standard 10 μL Tip. The scratched cells were washed off by PBS. Next, 1800 μL of New Medium and 20 g/L cactus extract was added. The appropriate distance of the gap was measured under the microscope, mark on the Dish, and take pictures at the same position at 0, 6, 12, 18, and 24 h, respectively.

### Dermatology skin patch test testing

This skin testing study procedure strictly followed the Good Clinical Practices and the Declaration of Helsinki and agreed with applicable institutional review board (IRB) regulations. Furthermore, the protocol corresponded to the “Technical standard for Cosmetic Human Skin Patch Testing (No. 1081603512)” by the Taiwan Food and Drug Administration and was approved by the IRB of Taichung Jen Ai Hospital, Taichung, Taiwan (approval certificate no. 110-12; approval date: 28/05/2021).

About 32 students from the Department of Medicinal Botanicals and Health Applications, Dayeh University (Changhua, Taiwan) were interviewed, and 15 were selected to participate in the single-application closed-patch epicutaneous test under semi-occlusion conditions. Table 1 shows the composition of the cactus extract test formulation. The carbopol gel was prepared with hot water. Next, the cactus extract, glycerin, TEA, and marine collagen were added and stirred vigorously. The test sample was freshly prepared before the beginning of the test. Participants were required to give their signed informed consent to be in the study. They completed the personal data form. Skin properties, such as the pore size, phlogosis, texture, oil, and moisture level were analyzed by using the skin analyzer (deViso skin analyzer, Prismatique, FL, USA) at the beginning of the test. Next, 1 mL of the test sample was dipped into the cotton pad and placed on the inner wrist. The control sample had sterilized water replaced on the wrist instead of cactus extract. After 10 min, the skin properties were analyzed by using the skin analyzer.

**Table 1:** Composition of formulation.

Composition	Percentage (%)
Water	90.0
Cactus extract	5.0
Glycerin	4.0
Carbopol	0.5
Foaming agent (TEA)	0.4
Marine collagen	0.1

### Statistical analysis

All experimental data are demonstrated as the mean ± standard deviation (SD) values. Student’s *t* test was applied to analyze the statistical analysis for multiple comparisons. A value of *P* < 0.05, and *P* < 0.01 was defined as statistically significant as compared with a control group.

### Statement of ethics

Written informed consent was obtained from all the participants involved in this study.

### Research involving plants

All procedures were conducted in accordance to the guidelines.

## Results

### Toxicity test of the cactus extract on B16-F10 cells

**Figure 2:**
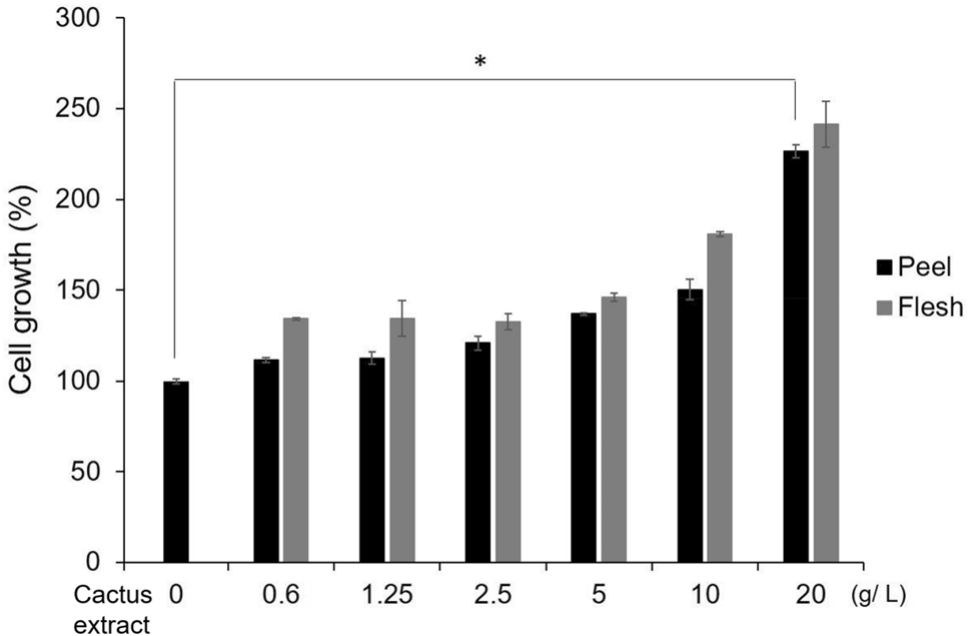
MTT analysis of cactus extract (peel and flesh) on cell growth. All data are presented as the mean ± S.D. of three independent experiments. *P < 0.05, **P < 0.01 compared with control group.

MTT assay was used to assess the safety of the cactus extract at 0.6 to 20 g/L for cell viability and proliferation of IBMX-induced B16-F10 melanoma cells. The peel and flesh of cactus extract had viability > 95% after treatments and no cytotoxic effects on B16-F10 cells (Fig. 2). From 0.6 to 20 g/L concentration, the cell growth was increased from 111 to 230% for peel extract and from 133 to 240% for flesh extract. The cell proliferation rate depended on the extract. Among the concentrations tested, 20 g/L of cactus extract had the most significant effect on cell proliferation, so cactus extract may promote the proliferation of B16-F10 cells.

### Ultraviolet protective effect of the cactus extract on B16-F10 cells

**Figure 3:**
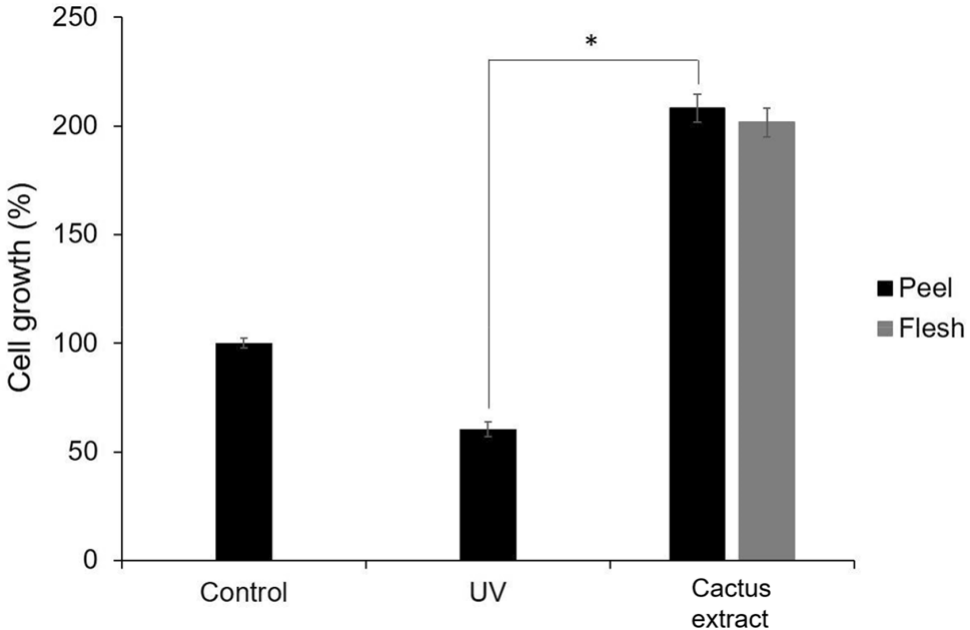
Ultraviolet protective effect of cactus extract (peel and flesh) on B16-F10 cells by MTT assay. All data are presented as the mean ± S.D. of three independent experiments. *P < 0.05, **P < 0.01 compared with control group.

We assessed the protective effect of adding 20 g/L cactus extract to B16-F10 cells under UV light exposure by MTT assay. B16-F10 cells with 20 g/L cactus extract that were irradiated with UV light for 1 h, then cultured for 24 h had higher survival than the control (100% cell growth) and UV-treated group (61% cell growth), which was 208% with peel extract and 200% with flesh extract, with no significant difference between peel and flesh extract (Fig. 3). The cactus extract could slow down the UV light irradiation damage of B16-F10 cells, with a marked difference in the effect of cell viability between the peel and flesh extract. Therefore, flesh extract was selected to continue the further whitening potential evaluation.

### Effects of the cactus extract on the expression of tyrosinase mRNA in B16-F10 cells

**Figure 4:**
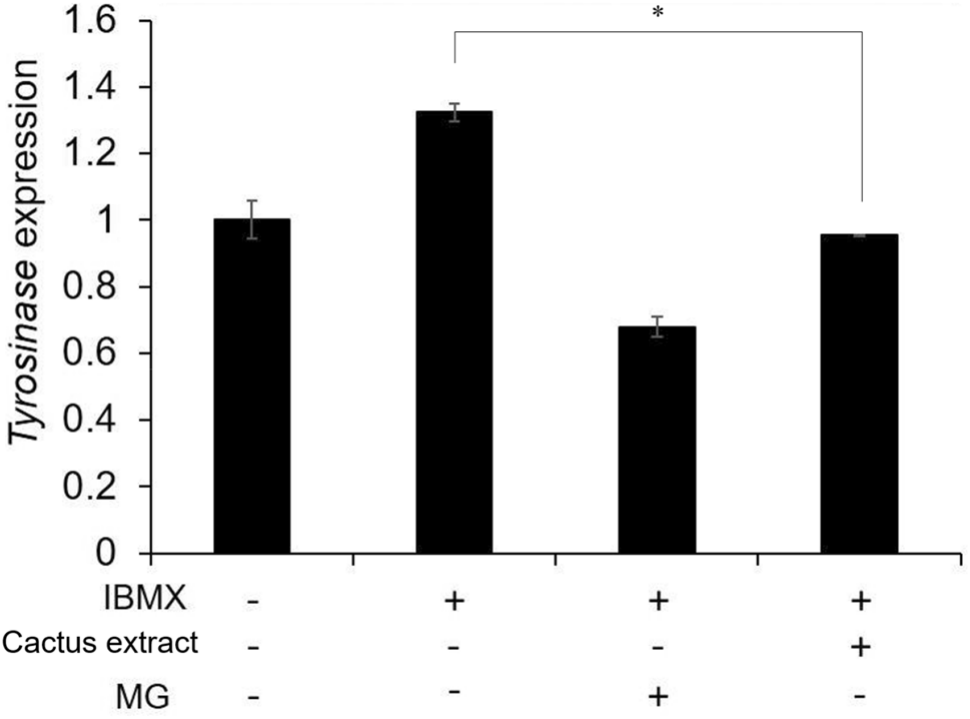
The effect of cactus extract (flesh) on tyrosinase mRNA expression in B16-F10 cells. All data are presented as the mean ± S.D. of three independent experiments. *P < 0.05, **P < 0.01 compared with control group.

The IBMX was applied to stimulate melanogenesis, which is associated with increased tyrosinase activity. Moreover, methyl gallate (MG) acts as a tyrosinase inhibitor that occurred naturally and was used as a positive control treatment in this study. The result showed that after treatment with MG, the tyrosinase expression of IBMX-induced B16-F10 cell was reduced to 0.66. The results in Fig. 4 also exhibited that after adding the cactus extract (flesh) of 20 g/L, the tyrosinase mRNA expression in cells decreased by 28% from 1.3 to 0.94, indicating that the cactus extract inhibited the expression of tyrosinase mRNA. Thus, the cactus extract was shown to function as a potential inhibitor of IBX-stimulated tyrosinase production in B16-F10 melanoma cells.

### Analysis of cactus extract for melanin production

**Figure 5:**
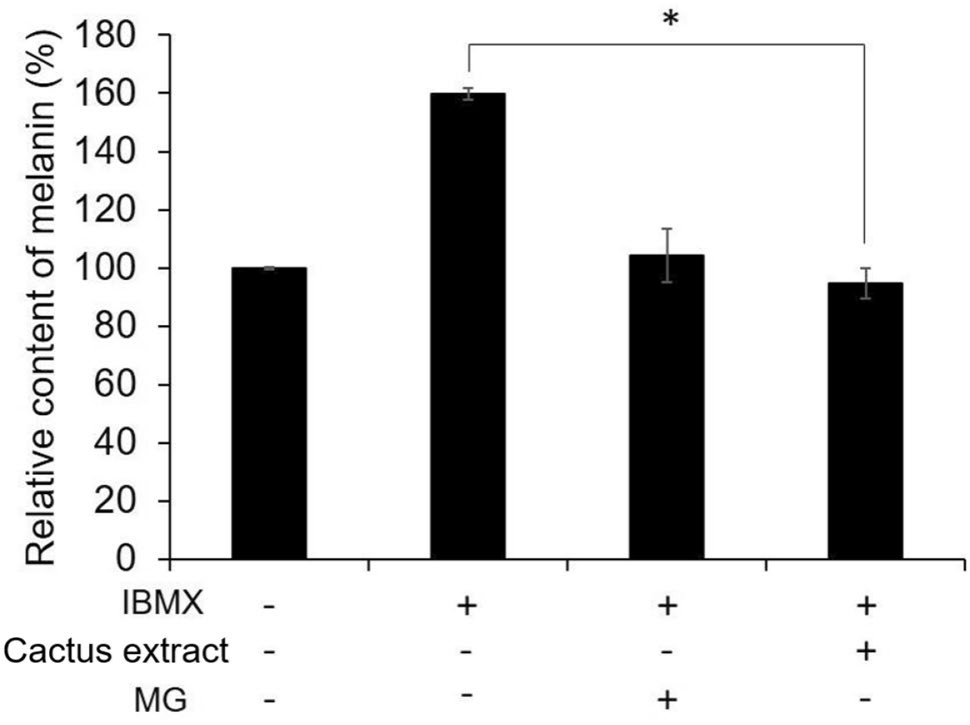
The effect of cactus extract (flesh) on melanin production content in B16-F10 cells. All data are presented as the mean ± S.D. of three independent experiments. *P < 0.05, **P < 0.01 compared with control group.

B16-F10 melanoma cells treated with 20 g/L to evaluate the quantity of melanin content was established in Fig. 5. The results indicated that the content of melanin in B16-F10 cells was reduced was 160% to 93%, which is 42%, after adding 20 g/L of the cactus extract. Nevertheless, the MG-treated IBMX-stimulated B16-F10 cell was only reduced to 102% of melanin content. Therefore, the cactus extract has the effect of reducing the production of melanin.

### The effect of the cactus extract on the growth rate of B16-F10 cells

**Figure 6:**
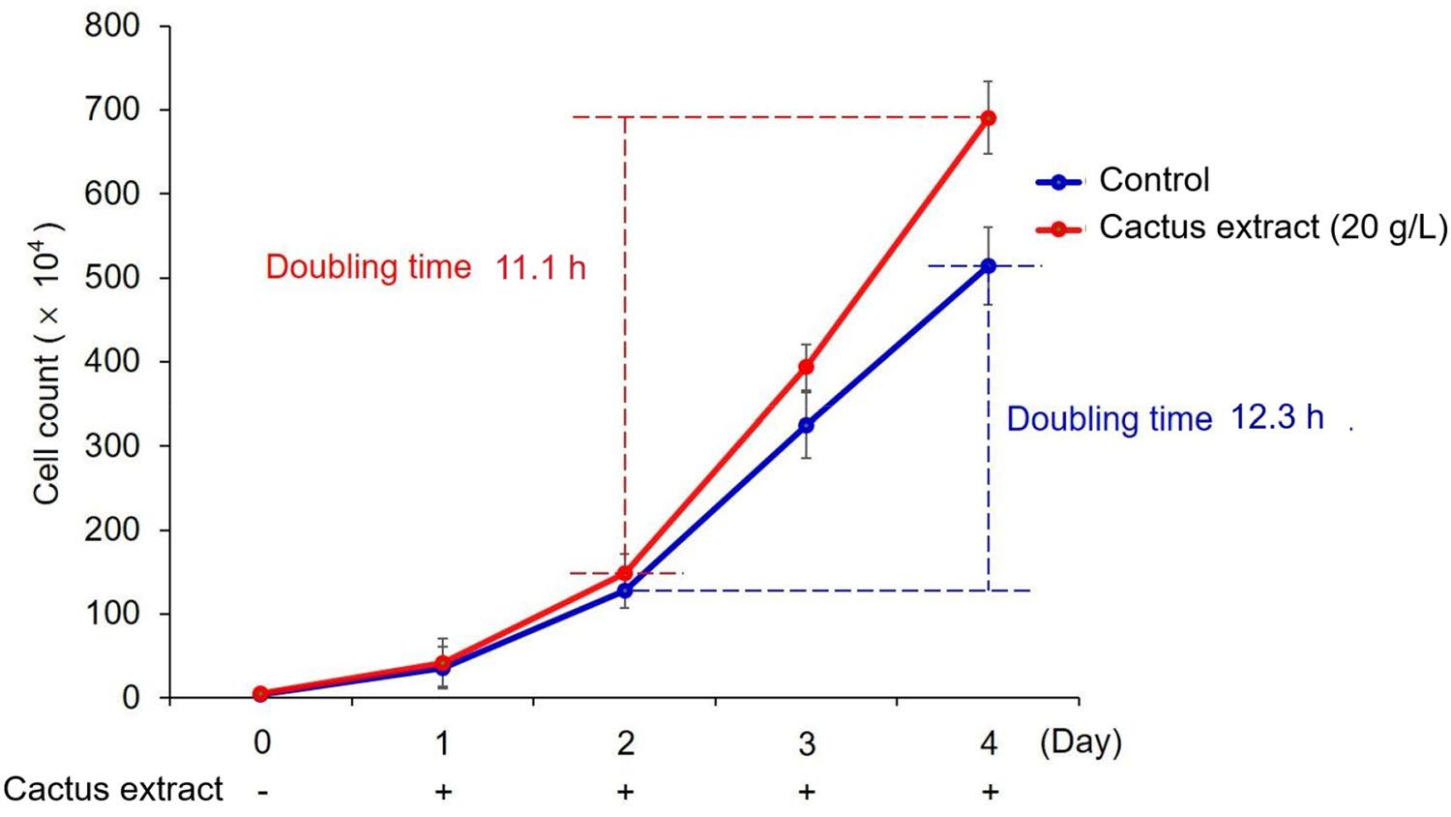
Cell growth rate analysis of cactus extract (flesh) with B16-F10 cells. All data are presented as the mean ± S.D. of three independent experiments.

After adding the cactus extract, the cell doubling time was 11.1 h as compared with 12.3 h for control, so cactus extract could accelerate cell growth (Fig. 6). Hence, the cactus extracts effectively reduced the doubling time and promoted cell growth.

### Effects of the cactus extract on growth factors of B16-F10 cells

**Figure 7:**
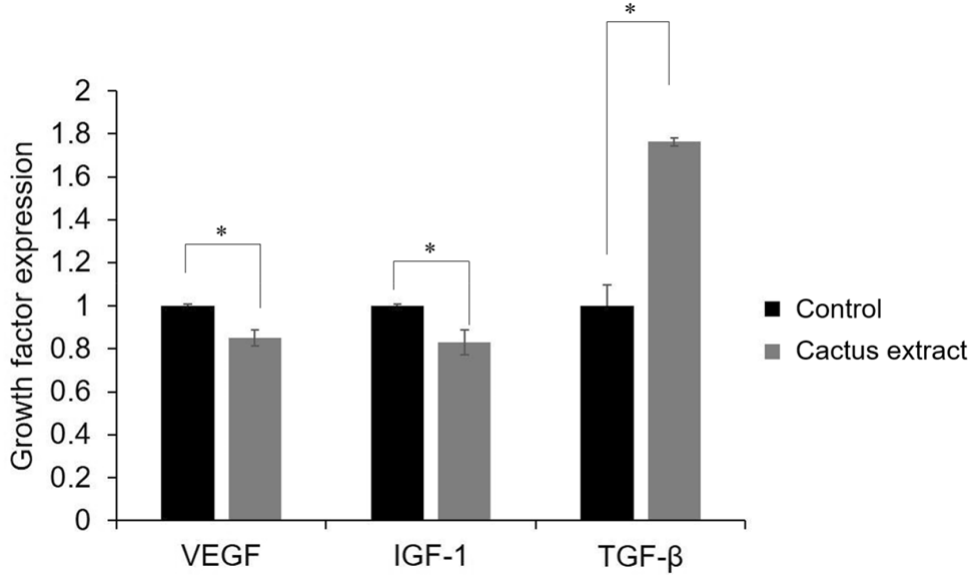
Effect of cactus extract (flesh) on growth factors of IBMX-induced B16-F10 cells. All data are presented as the mean ± S.D. of three independent experiments.

Figure 7 demonstrated the effect of the cactus extract on growth factors of IBMX-induced B16-F10 cells. The results presented that the expression of vascular endothelial growth factor (VEGF) and insulin-like growth factor (IGF-1) mRNA decreased from 1.02 ± 0.16 to 0.82 ± 0.13, and 1.05 ± 0.13 to 0.81 ± 0.10, respectively, after the addition of cactus extract of 20 g/L, which helped to prevent tumor proliferation and expansion; However, the expression level of transforming growth factor (TGF-β) mRNA has increased from 0.98 ± 0.13 (control) to 1.77 ± 0.11 (cactus extract). Thus, the cactus extract may have the ability to promote cell growth and wound healing.

### The effect of the cactus extract on the wound healing ability of B16-F10 cells

**Figure 8:**
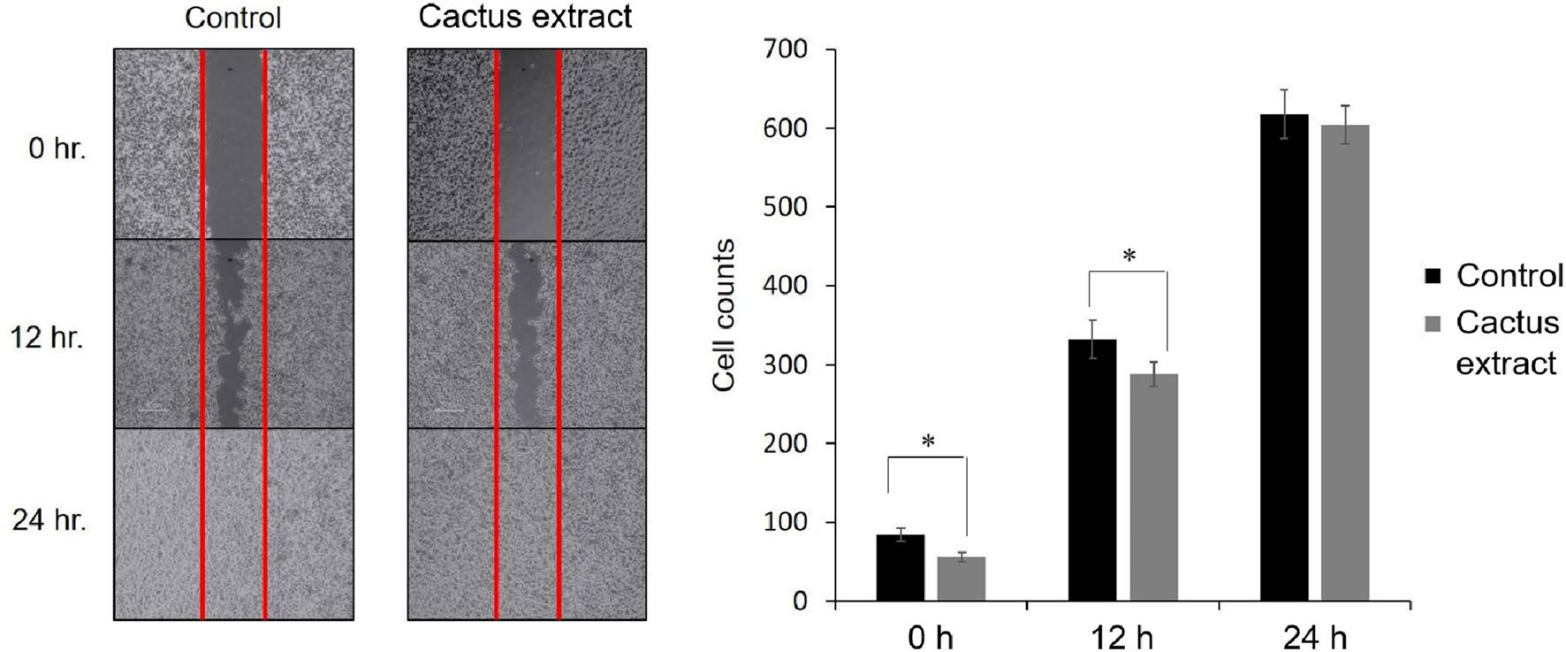
Wound healing assay to determine cell migration treated with cactus extract (flesh) by B16-F10 cells.

The migration ability of the cells treated with cactus extract differed as compared with controls (Fig. 8); thus, the cactus extract could promote the migration and spread of cancer cells. The cell counts of B16-F10 cells treated with cactus extract were increased from 54 ± 0.12 to 290 ± 0.08 and 602 ± 0.15 at 0, 12, and 24 h as compared with the control, which increased from 78 ± 0.06 to 342 ± 0.08 and 627 ± 0.17, respectively.

### Dermatology test

**Figure 9:**
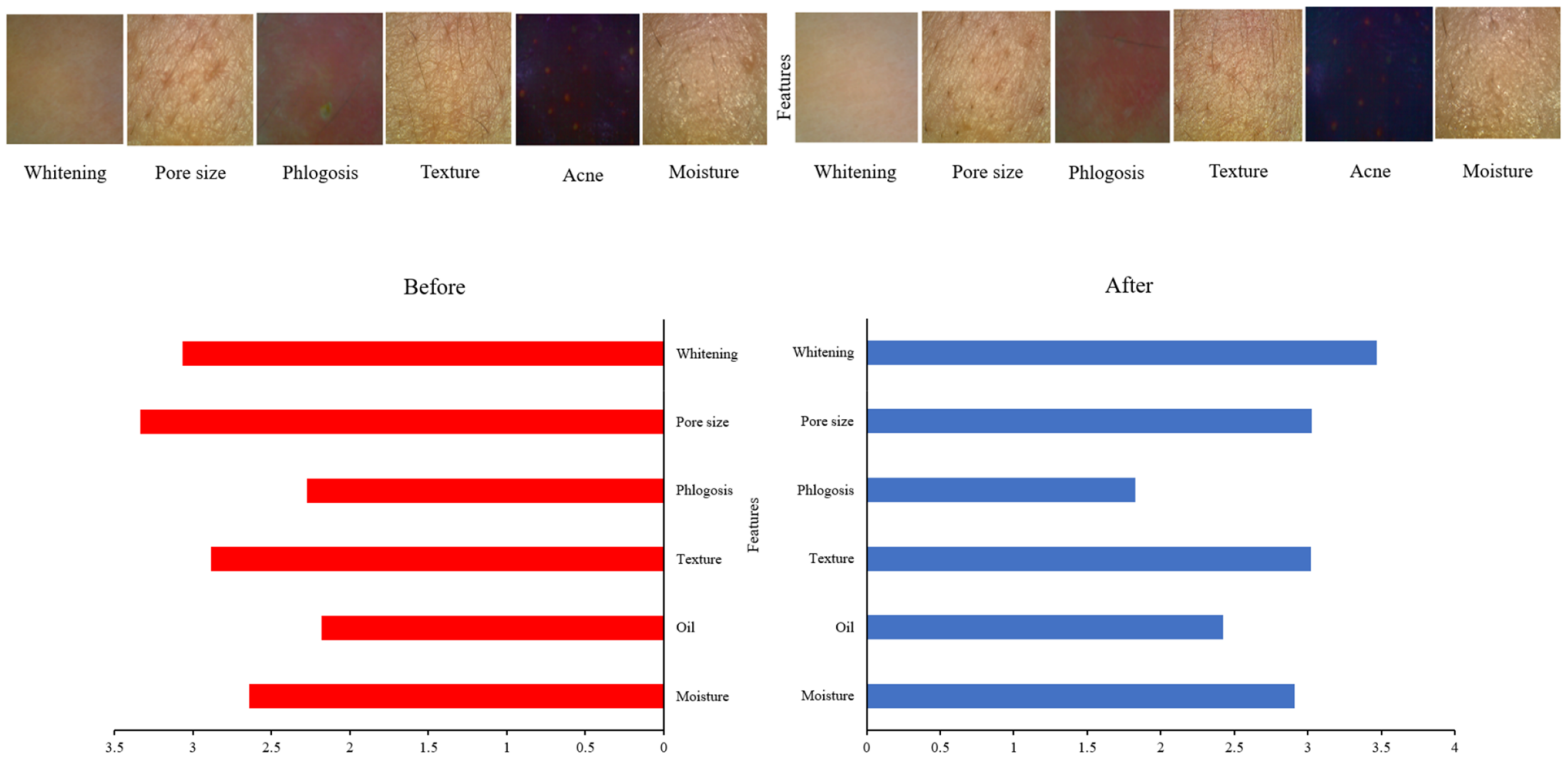
The whitening effect, pore size, phlogosis, texture, oil, and moisture level of skin before and after application of cactus extract.

Figure 9 concluded the dermatology skin patch test before and after applying the cactus extract sample. Before applying the test sample, the skin condition of each participant was recorded. After applying the test sample, the skin condition was analyzed for the second time to compare the effect before and after. The result demonstrated that the texture of the skin, oil, and moisture level of skin was improved after applying the test sample. In the meantime, the average pore size, and phlogosis situation decreased. It also can be observed from Fig. 9 that when the sample treatment was 10 min, the skin whitening effect was significantly raised from 3.065 to 3.467.

## Discussions

**Figure 10:**
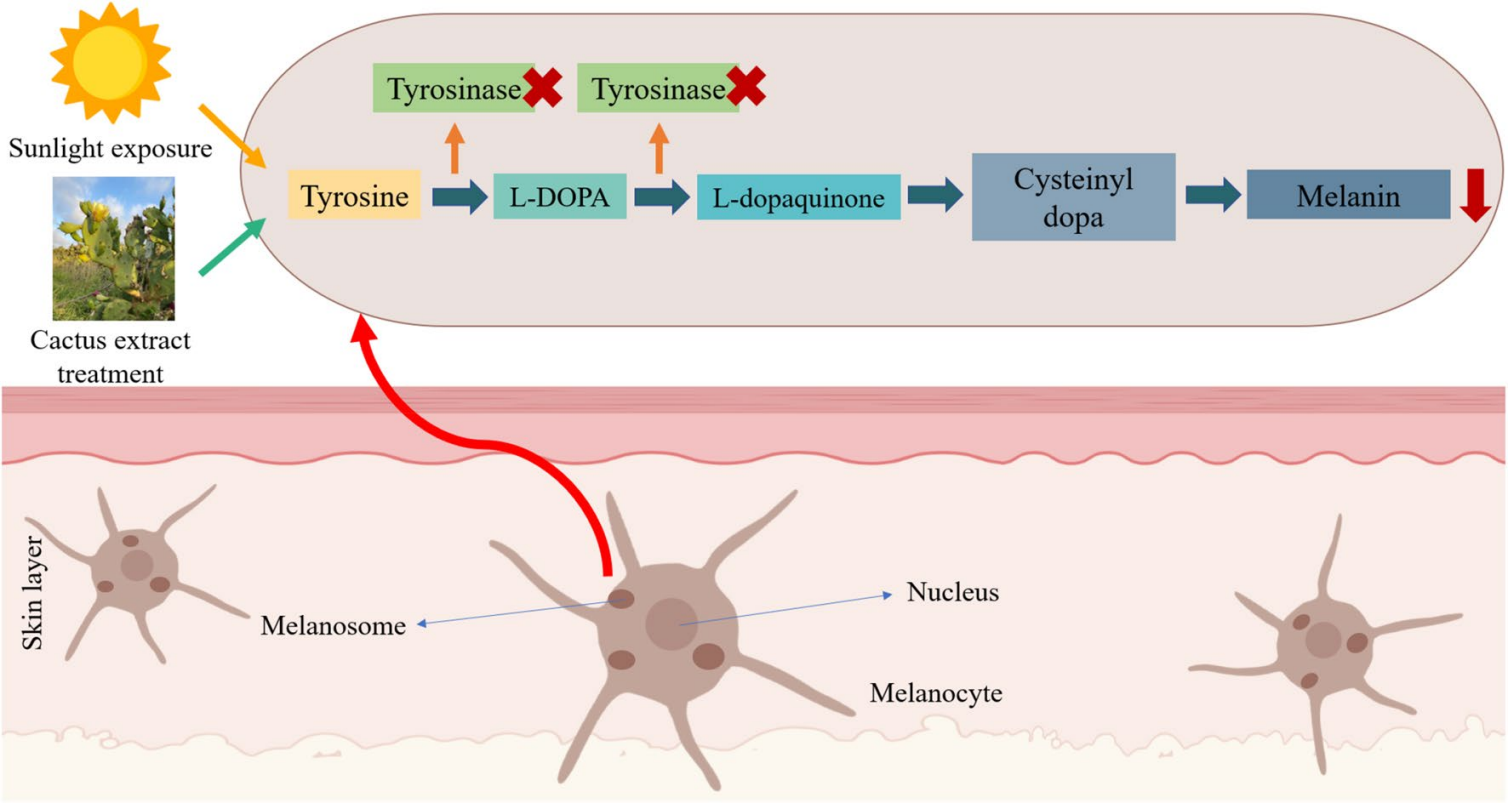
Inhibitory effects and mechanism of cactus extract on melanin production.

TO protect from strong sunlight exposure and prevent sunburn, melanin is the most crucial pigment in human skin. Melanin is usually produced by melanocytes in the epidermis layer through a biochemical synthesis called Raper-Mason pathway (RMP) of melanogenesis, and it will cause skin darkening^24^. The B16-F10 murine melanoma cells were not only used as a model for human skin cancers^25^, nevertheless, due to the characteristic of promoting melanogenesis after being stimulated, hence, B16-F10 cell line was selected in this study. The result of this study concluded that the cactus extract effectively cured the B16-F10 cells after damage by UV light irradiation (Fig. 3). Meanwhile, the cactus extracts were also applied oneself on preventing tyrosinase mRNA expression (Fig. 4) and melanin synthesis (Fig. 5) in IBMX-treated B16-F10 melanoma cells (Fig. 10). The result was corresponding to the previous study that concluded that *Opuntia humifusa* cladode extract reduced the enzyme gene (hyaluronidase mRNA) expression against UVB-induced skin degeneration^26^.

In this study, the results revealed that cactus extract significantly increased cell growth (Fig. 2) with a doubling time of 11.1 h (Fig. 6) by decreasing the VEGF and IGF-1, in the meantime, increased the TGF-β (Fig. 7). According to the results presented in the previous study, some of the integral constituents of *O. dillenii* cladodes, for example, the α-pyrones, opuntiol, and opuntioside, reduced the pain effectively and showed an analgesic response^19^. The cytotoxic effect of *O. dillenii* extracts had been studied previously by using the human breast cancer cells (MCF-7), human colon cancer cells (LoVo), and human liver hepatocytes (HepG2), and the results revealed that only low cytotoxicity and significant DNA-protective effects which mainly due to the phenolic compounds, terpenoids and fatty acid derivatives^27^.

After an injury, the primary inflammatory responses will trigger the proliferative phase to begin^28^. The re-epithelialization, angiogenesis, and fibroplasia are the main and leading events that happen during this phase^29^. Macrophage-released cytokines, for example, the TGF-β, FGF, and vascular EGF (VEGF) will promote angiogenesis and modulate endothelial cell proliferation^30^. VEGF and receptors play a vital role in regulating angiogenesis and vascular permeability^31^. VEGF-A binds and activates two tyrosine kinase receptors, VEGFR (VEGF receptor)-1 and VEGFR-2, and ultimately leads to angiogenesis, which is important in tumorigenesis^32^. When tumor tissue was under a hypoxic environment, Hypoxia-Inducible-Factor-1 (HIF-1) will activate some genes that contribute to angiogenesis, including VEGF, and promote vascular permeability^33,34^. Besides, insulin-like growth factor-1 (IGF-1), also known as somatomedin C, is one of the polypeptide hormones that regulate growth-promoting growth hormone (GH). This GH significantly affects the growth of cells, especially during cell division, to regulate the cells that can pass through the G1 phase and enter the S phase. When IGF-1 binds to the IGF-1 receptor, IGF-1 phosphorylated the tyrosine on the receptor, triggering intracellular signaling, which can activate the AKT signaling pathway and the Ras signaling pathway, resulting in cell growth and proliferation, in the meantime, inhibiting the programmed cell death^35^.

The result of this study was corresponding to the previous study which indicated that the lignin extract from *O. fícus-indica* and *O. cochenillifera* were capable to induce high cell proliferation during experimental times^36^. In addition, oil extract of *O. fícus-indica* from Tunisia not only revealed a significant antimicrobial, anti-yeast, and antifungal effect in vitro against bacteria, yeast, and fungi, meanwhile, also demonstrated a good wound healing effect with short scarring time in rat wound healing model^37^. Nevertheless, the bioactive profile generally altered with species, cultivar, climate, and growing condition, hence, further investigation needs to carry out to study the active components of *Opuntia dillenii* (Ker.) Haw.

For the dermatology skin patch test, the result of this study presented that the cactus extracts significantly enhanced the whitening, texture, oil, and moisture of the skin (Fig. 9), meanwhile, effectively decreasing the pore size, and phlogosis of the skin. The dermatology skin patch test result was in accordance with the result of in vitro study by using the B16-F10 melanoma cells. The cactus extract significantly inhibited the tyrosinase activity and resulted in lower melanin produced, in the meantime, showing the whitening effect in the dermatology skin test. Increasing moisture content means an upsurge in the moisture content of stratum corneum, and skin metabolism function^38^. Excessive skin grease secretion, no complete skin cleaning, blocking pores, and skin inflammation will cause the lipid of the skin level even worse. Additionally, the changes in moisture and oil level will influence the texture of the skin^39^. Likewise, the pore size was to analyze whether the skin absorption, secretion, excretion, and metabolic of pores are in good condition. Also, the phlogosis analysis was to examine skin sensitivity, such as skin redness, pyrexia, itchy, and pain^40^.

In this experiment, we explored the effect of adding the cactus extract to the B16-F10 cell line. A 20 g/L amount of cactus extract greatly suppressed IBMX-induced melanogenesis in B16-F10 cells. Adding 20 g/L cactus extract to B16-F10 cells triggered cell proliferation. After the addition of cactus extract, the mRNA expression of VEGF and IGF-1 decreased, which helped to prevent tumor proliferation and swelling. In short, cactus extract can promote cell growth and wound healing. However, the mRNA expression of TGF-β increased. Therefore, the CCD966SK cell line should be used to further study the ability of the cactus extract to heal wounds and reduce wrinkles.

Cactus extract greatly weakened melanin production by inhibiting tyrosinase activity in IBMX-stimulated B16-F10 cells. The mRNA expression of tyrosinase in B16-F10 cells decreased with 20 g/L of cactus extract and adding the cactus extract could effectively reduce B16-F10 cells producing melanin. Moreover, the survival rate of the cells was improved on exposure to UV light, so the cactus extract could protect cells against UV light damage and inhibit the production of melanin.

## Conclusion

*O. dillenii* cactus extract can increase the growth rate of cells by affecting growth factors and can protect cells against UV light damage and inhibit melanin production. The cactus extract contains multiple protective effects and deserves further research and development as a skin care product. Moreover, the effects of the cactus extract on the Raper-Mason pathway require further study.

## Data Availability

The datasets used during the current study available from the corresponding author on reasonable request.
